# Choosing the Optimal Spatial Domain Measure of Enhancement for Mammogram Images

**DOI:** 10.1155/2014/937849

**Published:** 2014-08-06

**Authors:** Karen Panetta, Arash Samani, Sos Agaian

**Affiliations:** ^1^Department of Electrical and Computer Engineering, Tufts University, Medford, MA, USA; ^2^Department of Electrical and Computer Engineering, University of Texas at San Antonio, San Antonio, TX, USA

## Abstract

Medical imaging systems often require image enhancement, such as improving the image contrast, to provide medical professionals with the best visual image quality. This helps in anomaly detection and diagnosis. Most enhancement algorithms are iterative processes that require many parameters be selected. Poor or nonoptimal parameter selection can have a negative effect on the enhancement process. In this paper, a quantitative metric for measuring the image quality is used to select the optimal operating parameters for the enhancement algorithms. A variety of measures evaluating the quality of an image enhancement will be presented along with each measure's basis for analysis, namely, on image content and image attributes. We also provide guidelines for systematically choosing the proper measure of image quality for medical images.

## 1. Introduction

Mammography is noninvasive imaging that uses a low-dose X-ray to photograph breast tissue. The result of mammography is a mammogram, which is used as a screening test for breast cancer. Mammography is a valuable screening procedure that can detect breast cancer early, as long as two years before a lump can be felt. Mammography also is used to help clarify whether a suspicious breast lump is a cyst or a tumor and whether a tumor is more likely to be benign or malignant. Mammography misses breast cancer about 5% to 10% of the time, but the rate can be as high as 30% for women with dense breast tissue [1]. The X-ray hardware limitation and the high density breast tissue will affect the quality of mammograms that can contribute to misdiagnosis of breast cancer. Enhancing the contrast of mammogram images could improve the results of mammography significantly [2, 3].

There are many different techniques for enhancing the quality of an image [2–7], which allow the observer to better perceive the desirable information in the image. These techniques often have parameters to control the enhancement process and its outcome. To optimize these parameters, feedback is required in the enhancement process, which is a quantitative assessment of image quality, known as measure of enhancement or image quality measure. There have been different definitions of an adequate measure of performance based on contrast [8–10]. Contrast based measure of enhancement methods can be categorized as either spatial or transform based domain measures [10]. The spatial domain measures are calculated based on luminance of pixels in different portions of an image, but the transform domain measures work based on the Discrete Cosine Transform (DCT), Discrete Fourier Transform (DFT), or Discrete Wavelet Transform (DWT) of the image [11]. Most spatial domain measures are derivatives of the Weber-Fechner law, Michelson contrast measure [12], or Contrast Ratio (CR), using statistical analysis to better evaluate the image contrast enhancement. EME, EMEE, AME, AMEE, LogAME, and LogAMEE are examples of such spatial domain measures [4, 5, 13].

Performance of spatial domain measures is highly dependent on image attributes such as image content, lighting, uniform versus nonuniform background, texture, periodic patterns, randomness, single versus multiple targets, noise, and distortions. The choice of a measure of enhancement method could affect the outcome of image enhancement algorithms. If a measure is not designed to handle a specific image attribute properly, that measure will not provide a good metric for properly evaluating the contrast enhancement for that specific image.

In this paper, we explain the relationship between the image properties and the commonly used spatial domain measures of image quality. We will also provide guidelines of how to choose the measure of enhancement based on the image attributes and how to select the optimal operating parameters that are used by these measures. Finally, recommendations are presented for determining which spatial domain measure is best suited to assess the quality of mammogram images.

## 2. Materials and Methods

When enhancing a digital image, there is a need for a quantitative measure to evaluate the visual performance of the enhancement process. In iterative automated enhancement algorithms, one goal of the enhancement measure is to provide a statistic to help optimize the enhancement algorithm's parameters. These measures are based on luminance contrast statistics of the image and generally have two components. The first is comparing the difference between the two luminance components in question (e.g., of a symbol and its background). If the state of adaptation of the visual system stays constant, larger luminance differences produce larger brightness differences (higher brightness contrast). The second component of any luminance contrast statistic is some measure describing the adaptation state of the eye. A luminance that produces a large brightness on a dim background will produce a smaller brightness difference on a brighter background due to visual adaptation. To capture this behavior, designers of luminance contrast statistics generally divide a numerator that describes the luminance change by a denominator that describes the average luminance to which the eye is adapted:

(1) $$\begin{array}{c} \text{Luminance}\;\text{Contrast}=\frac{\text{Luminance}\;\text{Change}}{\text{Adaptation}\;\text{Descriptor}}. \end{array}$$

The variety of popular statistics for luminance contrast mostly reflects the fact that the adaptation state of the eye is affected differently by different kinds of stimulus patterns. In the following section, we provide the fundamental measures used to evaluate contrast.

### 2.1. Basic Measures of Image Contrast

#### 2.1.1. Michelson Contrast

This measure is commonly used for patterns where both bright and dark features are equivalent and cover similar fractions of the area, for example, periodic patterns such as a sinusoidal grating. Michelson contrast is defined as

(2) $$\begin{array}{c} C_{M}=\frac{I_{\max}-I_{\min}}{I_{\max}+I_{\min}} \end{array}$$

with *I*_max⁡_ and *I*_min⁡_ representing the highest and lowest luminance. The denominator represents twice the average of the luminance [12]. For simple periodic patterns (e.g., textures) there is no large area of uniform luminance that dominates the user's brightness adaptation. The denominator of C_M_ is twice the mean of the maximum and minimum luminance; that is, the adaptation luminance estimate is based on the space-average luminance.

#### 2.1.2. Weber-Fechner Law

The Weber-Fechner law implies a logarithmic relationship between physical luminance and subjectively perceived brightness. Weber contrast measure assumes a large uniform luminance background with a small test target:

(3) $$\begin{array}{c} C_{W}=\frac{I_{s}-I_{b}}{I_{b}}, \end{array}$$

where *I*_*s*_ is the luminance of the target and *I*_*b*_ is the luminance of the immediately adjacent background. It is commonly used in cases where small features are present on a large uniform background; that is, the average luminance is approximately equal to the background luminance. When the background is lighter than the target *C*_*W*_ is negative and ranges from zero to −1. When the background is darker than the target *C*_*W*_ is positive and ranges from zero to potentially very large numbers.

The Weber-Fechner measure is used in cases where the average luminance is approximately equal to the background luminance.

#### 2.1.3. Contrast Ratio

This measure has often been applied to the stimulus class in either linear or logarithmic form:

(4) $$\begin{array}{c} CR=\frac{I_{s}}{I_{b}},\;\;\log\left(CR\right)=\frac{\log\left(I_{s}\right)}{\log\left(I_{b}\right)}. \end{array}$$

This measure, *C*_*R*_, does not mathematically compare with the Weber contrast since the numerator is not the luminance difference between the target and the background.

#### 2.1.4. Entropy

Entropy is calculated from the histogram of an image and is calculated over the entire image. It is a scalar value representing the entropy of an intensity image, a statistical measure of randomness that can be used to characterize the texture of the image:

(5) $$\begin{array}{c} \text{Entropy}=-\sum p*\ln\left(p\right), \end{array}$$

where *p* is the histogram count for a segment of image.

Since entropy is calculated over the entire image, rearranging segments of the image would not change this contrast measure. Also increasing the contrast in one part of an image and decreasing it in another part may result in similar entropy as the original image.

### 2.2. Complex Measures of Contrast

**Figure 1:**
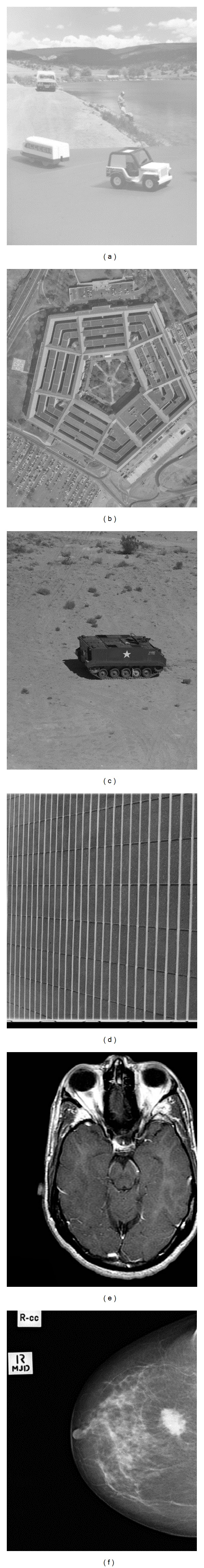
Examples of different image attributes. (a) Large uniform background, small targets, no randomness, and no peridic patterns; (b) semiperiodic, nonuniform background; (c) large uniform background, textured in upper part of image, nonperiodic; (d) periodic, nonuniform background; and ((e), (f)) random texture, nonpediodic, nonuniform background.

These measures are based on the basic contrast measures with additional optimization parameters. Since the basic measures of contrast are highly sensitive to image contents and attributes such as noise, periodicity, texture, randomness, uniform background, and target size, often a combination of two basic measures in a new quality measure could extend the application of this new complex measure. The image contents should be visually inspected to choose the optimal measure.  Figure 1 shows examples of different image attributes.

#### 2.2.1. EME

Let an image *x*(*n*, *m*) be split into *k*_1_ *k*_2_ blocks of *W*_*k*,*l*_(*i*, *j*); the EME is defined as

(6) $$\begin{array}{c} {\text{EME}}_{k_{1}k_{2}}=\frac{1}{k_{1}k_{2}}\overset{k_{1}}{\underset{l=1}{\sum }}\;\overset{k_{2}}{\underset{k=1}{\sum }}\left[20\ln\left(\frac{I_{\max;k,l}}{I_{\min;k,l\;}}\right)\right], \end{array}$$

where *I*_min⁡;*k*,*l*_ and *I*_max⁡;*k*,*l*_ are the minimum and maximum of the image inside the block *W*_*k*,*l*_. As defined in (4), for each block of (*k*, *l*) in (*k*_1_, *k*_2_) blocks, the Contrast Ratio is

(7) $$\begin{array}{c} {CR}_{k,l}=\frac{I_{\max;k,l}}{I_{\min;k,l\;}}. \end{array}$$

If *k*_1_ *k*_2_ = 1 (image is divided into one block), this method will return result similar to Contrast Ratio (*C*_*R*_):

(8) $$\begin{array}{c} {\text{EME}}_{k_{1}k_{2}}=\frac{1}{k_{1}k_{2}}\overset{k_{1}}{\underset{l=1}{\sum }}\;\overset{k_{2}}{\underset{k=1}{\sum }}\left[20\ln\left({CR}_{k,l}\right)\right]. \end{array}$$

Dividing the image into *k*_1_ *k*_2_ blocks will turn the complex image into simple blocks assuming that there is only one target per block and *I*_*S*_ turns out to be *I*_max⁡_ and the background (*I*_*b*_) carries *I*_min⁡_.

The EME measure of enhancement is suitable for images with the following properties:

- noncomplex segments (CR property);
- uniform background in segments (Weber property);
- small targets in segments (Weber property);
- nonperiodic pattern in segments (Weber property);
- little to no randomness in segments (no entropy in measure).

The EME measure would not be a good choice for images with these attributes:

- complex segments within an image or complex images with large block size (CR property);
- images with nonuniform background in each segment or images with large segment size (Weber property);
- large target in segments the way that the segment is equally divided into high and low luminance areas; this attribute also extends to periodic patterns and images with random texture (Weber property).

#### 2.2.2. EMEE

Let an image *x*(*n*, *m*) be split into *k*_1_ *k*_2_ blocks of *W*_*k*,*l*_(*i*, *j*); the EMEE is defined as

(9) $$\begin{array}{c} {\text{EMEE}}_{{\alpha k}_{1}k_{2}}=\frac{1}{k_{1}k_{2}}\overset{k_{1}}{\underset{l=1}{\sum }}\;\overset{k_{2}}{\underset{k=1}{\sum }}\left[{\alpha \left(\frac{I_{\max;k,l}}{I_{\min;k,l\;}}\right)}^{\alpha }\ln\left(\frac{I_{\max;k,l}}{I_{\min;k,l\;}}\right)\right]. \end{array}$$

Similar to entropy (5), for each block *W*_*k*,*l*_ of the image, the ratio

(10) $$\begin{array}{c} p={\left(\frac{I_{\max;k,l}}{I_{\min;k,l\;}}\right)}^{\alpha } \end{array}$$

represents the number of intensity levels in that block if block is normalized by *I*_min⁡;*k*,*l*_ if *α* = 1. For each block, the Contrast Ratio is defined as in (7); therefore the EMEE measure yields to

(11) EMEEαk1k2=1k1k2∑l=1k1 ∑k=1k2[α(CRk,l)αln⁡(CRk,l)]=1k1k2∑l=1k1 ∑k=1k2[Entropy(CRα)].

Therefore, the EMEE is the entropy of the Contrast Ratio for each block *W*_*k*,*l*_ scaled by *α*, averaged over the entire image. This association makes EMEE measure of enhancement suitable for images with the following properties:

- noncomplex segments (CR property);
- nonperiodic patterns in segments (Weber property);
- being able to handle randomness in texture (because of added entropy compared to EME);
- using a larger “*α*” parameter will help to handle more randomness in image texture by emphasizing the entropy term.

EMEE measure is not a good choice to handle

- images with complex segments or if segment size is chosen too large to create a complex segment (CR property);
- periodic images or images where the high and low luminance are equally spread in segments (Weber property).

#### 2.2.3. AME

Let an image *x*(*n*, *m*) be split into *k*_1_ *k*_2_ blocks of *W*_*k*,*l*_(*i*, *j*); the AME is defined as

(12) $$\begin{array}{c} {\text{AME}}_{k_{1}k_{2}}=-\frac{1}{k_{1}k_{2}}\overset{k_{1}}{\underset{l=1}{\sum }}\;\overset{k_{2}}{\underset{k=1}{\sum }}\left[20\ln\left(\frac{I_{\max;k,l}-I_{\min;k,l\;}}{I_{\max;k,l}+I_{\min;k,l\;}}\right)\right]. \end{array}$$

From the definition of Michelson contrast in (2) we have

(13) $$\begin{array}{c} {\text{AME}}_{k_{1}k_{2}}=-\frac{1}{k_{1}k_{2}}\overset{k_{1}}{\underset{l=1}{\sum }}\;\overset{k_{2}}{\underset{k=1}{\sum }}\left[20\ln\left(C_{M,k,l}\right)\right]. \end{array}$$

This means that AME measure of enhancement for an image is an average of Michelson contrast for each block *W*_*k*,*l*_, in a logarithmic form, over the entire image. The AME measure is suitable for images with the following properties:

- periodic patterns in segments (Michelson property);
- no randomness in texture (lack of entropy).

This measure lacks ability to analyze images with the following attributes:

- images with uniform background (Michelson property);
- areas of large uniform luminance is segments (Michelson property);
- randomness in image texture (entropy property).

#### 2.2.4. AMEE

Let an image *x*(*n*, *m*) be split into *k*_1_ *k*_2_ blocks of *W*_*k*,*l*_(*i*, *j*); the AMEE is defined as

(14) AMEEαk1k2=−1k1k2 ×∑l=1k1 ∑k=1k2[α(Imax⁡;k,l−Imin⁡;k,l  Imax⁡;k,l+Imin⁡;k,l  )αln⁡(Imax⁡;k,l−Imin⁡;k,l  Imax⁡;k,l+Imin⁡;k,l  )].

Using the definition of Michelson contrast (2) and entropy (5) we have

(15) AMEEαk1k2=−1k1k2∑l=1k1 ∑k=1k2[α(CM,k,l)αln⁡(CM,k,l)]=1k1k2∑l=1k1 ∑k=1k2[Entropy(CM,k,lα)].

In comparison with the relationship between EMEE and EME methods, the AMEE is simply the entropy-base measure of AME. In other words, AMEE is the average of entropy of the Michelson law for each block *W*_*k*,*l*_ over the entire image, scaled by *α*.

The AMEE measure is suitable for images with the following properties:

- periodic patterns in segments (Michelson property);
- no area of large uniform luminance in segments (Michelson property);
- being able to handle additional randomness in texture (entropy).

The AMEE measure does not perform well for the images with the following properties:

- images with large uniform background (Michelson property).

#### 2.2.5. LogAME

Let an image *x*(*n*, *m*) be split into *k*_1_ *k*_2_ blocks of *W*_*k*,*l*_(*i*, *j*); the LogAME is defined as

(16) logAMEk1k2=1k1k2⊗∑l=1k1 ∑k=1k2[120⊗ln⁡(Imax⁡;k,l⊖Imin⁡;k,l  Imax⁡;k,l⊕Imin⁡;k,l  )].

This measure is similar to AME measure, based on Michelson contrast for each block *W*_*k*,*l*_, in a logarithmic form, over the entire image. However, in this measure the arithmetic operations (∗, +, and −) were replaced by the PLIP arithmetic operations ⊗, ⊕, and ⊖ [5]. The coefficient changes and the sign change are to provide a comparable numeric return to AME method. Using the log and the PLIP operations will put more emphasis on areas with low luminance.

If we define a version of Michelson contrast that uses the PLIP operators as

(17) $$\begin{array}{c} \text{Log}C_{M}=\;\frac{I_{\max}⊖I_{\min}}{I_{\max}⊕I_{\min}}, \end{array}$$

we can rewrite the LogAME measure as

(18) $$\begin{array}{c} {\text{logAME}}_{k_{1}k_{2}}=\frac{1}{k_{1}k_{2}}⊗\overset{k_{1}}{\underset{l=1}{\sum }}\;\overset{k_{2}}{\underset{k=1}{\sum }}\left[\frac{1}{20}⊗\ln\left({\text{Log}C}_{M,k,l}\right)\right]. \end{array}$$

The LogAME measure is suitable for images with the following properties:

- periodic patterns in segments (Michelson property);
- unlike AME, LogAME can better handle areas with large uniform luminance in blocks or between blocks (PLIP property).

For images with small differences between target and background luminance, LogAME will behave similarly to AME.

The LogAME measure will not be the best choice for measure of enhancement for images with the following attributes:

- images with small targets and a large background (Michelson property);
- images with small difference between the background luminance and the target luminance (PLIP property);
- images with randomness in texture (entropy property).

#### 2.2.6. LogAMEE

Let an image *x*(*n*, *m*) be split into *k*_1_ *k*_2_ blocks of *W*_*k*,*l*_(*i*, *j*); the LogAMEE is defined as

(19) logAMEEαk1k2  =1k1k2⊗∑l=1k1∑k=1k2[(Imax⁡;k,l⊖Imin⁡;k,l  Imax⁡;k,l⊕Imin⁡;k,l  )               ∗ln⁡(Imax⁡;k,l⊖Imin⁡;k,l  Imax⁡;k,l⊕Imin⁡;k,l  )].

This measure is similar to AMEE measure; it is an entropy-base measure of AME which is the average of entropy of the Michelson law for each block *W*_*k*,*l*_ over the entire image, but the arithmetic operations were replaced by the PLIP arithmetic operations. Using the log and the PLIP operations will put more emphasis on areas with low luminance.

Combining the same techniques that we used in LogAME and EMEE measures we get

(20) $$\begin{array}{c} {\text{logAMEE}}_{{\alpha k}_{1}k_{2}}=\frac{1}{k_{1}k_{2}}⊗\overset{k_{1}}{\underset{l=1}{\sum }}\overset{k_{2}}{\underset{k=1}{\sum }}\left[\text{entropy}\left({\text{Log}C}_{M,k,l}\right)\right]. \end{array}$$

The LogAMEE measure is suitable for images with the following properties:

- periodic patterns in segments (Michelson property);
- unlike AMEE, it can better handle areas with large uniform luminance in segments (PLIP property);
- in comparison with LogAME, it can handle additional randomness in texture (entropy property);
- for images with small differences between target and background luminance, LogAMEE will behave similarly to AMEE.

The LogAMEE measure is not a good measure for images with these properties:

- images with small targets and a large background (Michelson property);
- images with small difference between the background luminance and the target luminance (PLIP property).

### 2.3. Choosing the Measure for Mammograms

In a mammogram image, the large black area beside the breast is not considered the image background (the adaptation state of the eye); instead we consider the breast itself as the background and the malignant tissues, cysts, and calcifications as the target. Hence, the image does not include a large uniform background, which makes EME and EMEE measures inappropriate quality measures to use for these images. The lack of the presence of a large uniform background makes Michelson contrast base measures (AME, AMEE, LogAME, and LogAMEE) good candidates for mammograms. Considering the small difference between the luminance of breast tissue and the luminance of the area containing the abnormality, it is not recommended to use the LogAME and LogAMEE measures, which are best suited for images with the large luminance difference between the target and the background. Also, because of the display of soft tissues in X-ray images, mammograms tend to be textured and have a fair amount of randomness in the target area of the image. This attribute aligns with the properties of entropy-base measures, which can handle images with randomness and texture better. Therefore, considering the above argument about the nature of the mammograms, we recommend using AMEE measure of image quality when evaluating these images.

In the next section, we compare the performance of these measures on a database of mammogram images.

## 3. Results

**Figure 2:**
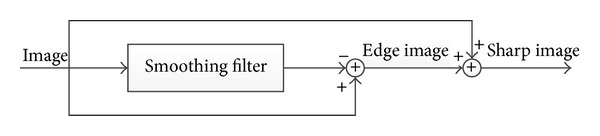
Unsharp masking contrast enhancement.

**Figure 3:**
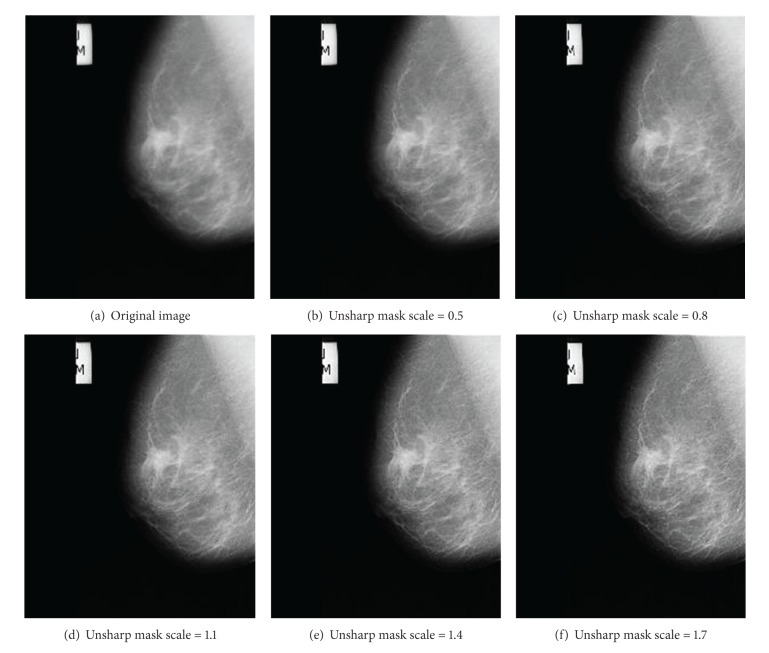
Image enhancement using unsharp masking. Higher unsharp mask scale results in higher contrast in enhanced image.

For our study, we used mammograms from “the mini-MIAS database of mammograms” [14]. To evaluate the performance of different measures, each mammogram was enhanced using unsharp masking contrast enhancement (Figure 2), alpha-rooting, CLAHE, and Lee's enhancement algorithm [15, 16]. As demonstrated in Figure 3, increasing the unsharp mask scale will result in enhancement of image contrast. We used this scale as the ground truth for studying the performance of different quality measures. The enhanced images from [14] were tested using different quality measures.

**Figure 4:**
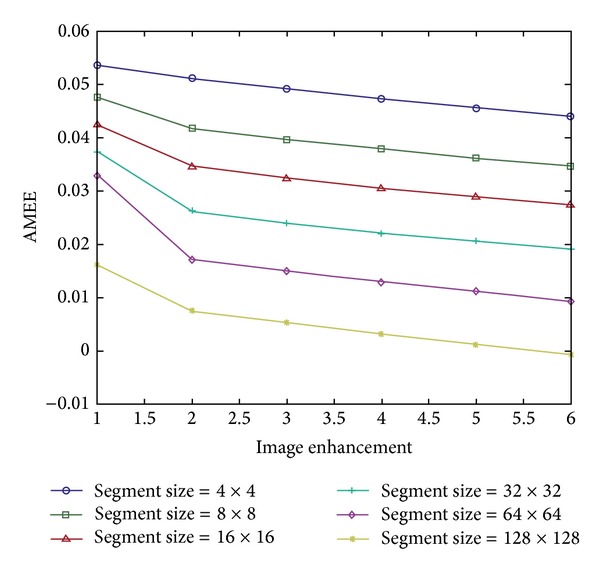
Enhanced image from Figure 3 was tested by AMEE measure. Higher image index (*x*-axis) means higher image contrast; also lower AMEE measure (*y*-axis) indicates more enhanced image.

Figure 4 shows an example of images from Figure 3 evaluated by the AME measure using different segment sizes. As the scaling constant increases, the image will have higher contrast. A lower AMEE measure also shows higher image contrast. For an optimal segment size, *k* > 16 × 16 pixels, a monotonic decrease in the AMEE indicates that the measure is correctly evaluating the image quality. Controlling the parameter *α* in the AMEE measure can help to better address *w* randomness in images. In our study, we set the parameter *α* to 0.1.

To quantify the overall performance of each quality measure over the entire MIAS database [14], we computed the Pearson correlation between each enhanced image and each quality measure and averaged over the entire database. There are 322 mammogram images in MIAS database [14] and we introduced 6 enhanced versions for each mammogram. For each measure of enhancement, we averaged the Pearson correlation for the 1932 images and results are shown in Table 1. When the average correlation is close to 1, it means that the measure and the enhancement are closely agreeing with each other, and a negative correlation indicates that the measure was not able to correctly evaluate the image enhancement. As shown in Table 1, the AMEE measure exhibits the best performance by predicting the image enhancement correctly in over 99.7% of images.

## 4. Conclusions

Like most medical imaging systems, mammography requires enhancement of low quality images due to the X-ray hardware limitations. Almost all image enhancement processes require reliable evaluation of the image quality to help with the parameter selection and optimization of enhancement process. In this study, we examined several image quality measures against a database of mammograms using different enhancement processes. We provided our recommendation for the optimal image quality assessment and its parameters for this category of images.

## Figures and Tables

**Table 1:** Average Pearson correlation for all 1932 images from MIAS database of mammograms (322 images with 6 enhanced versions of each image). The AMEE measure shows the best performance between the measures we tested.

Quality measure	EME	EMEE	AME	AMEE	LogAME	LogAMEE
Average correlation with enhancement	0.0781	0.0222	0.7854	0.9974	0.7851	0.3254

## References

[1] Rosenberg RD, Yankaskas BC, Abraham LA (2006). Performance benchmarks for screening mammography. *Radiology*.

[2] Yicong Z, Panetta K, Agaian S Human visual system based mammogram enhancement and analysis.

[3] Panetta K, Zhou Y, Agaian S, Jia H (2011). Nonlinear unsharp masking for mammogram enhancement. *IEEE Transactions on Information Technology in Biomedicine*.

[4] Agaian SS, Silver B, Panetta KA (2007). Transform coefficient histogram-based image enhancement algorithms using contrast entropy. *IEEE Transactions on Image Processing*.

[5] Panetta KA, Wharton EJ, Agaian SS (2008). Human visual system-based image enhancement and logarithmic contrast measure. *IEEE Transactions on Systems, Man, and Cybernetics, Part B: Cybernetics*.

[6] Silver B, Agaian S, Panetta K Contrast entropy based image enhancement and logarithmic transform coefficient histogram shifting.

[7] Wharton EJ, Panetta KA, Agaian SS, S. S. Agaian Human visual system based image enhancement.

[8] Chen G, Panetta K, Agaian S No reference color image quality measures.

[9] Panetta K, Chen G, Agaian S (2013). No reference color image contrast and quality measures. *IEEE Transactions on Consumer Electronics*.

[10] Samani A, Panetta K, Agaian S Transform domain measure of enhancement—TDME—for security imaging applications.

[11] Agaian SS, Panetta K, Grigoryan AM (2001). Transform-based image enhancement algorithms with performance measure. *IEEE Transactions on Image Processing*.

[12] Michelson AA (1927). *Studies in Optics*.

[13] Wharton E, Panetta K, Agaian S Human visual system based similarity metrics.

[14] Suckling J (1994). *The Mammographic Image Analysis Society Digital Mammogram Database Exerpta Medica*.

[15] Jong-Sen L (1980). Digital image enhancement and noise filtering by use of local statistics. *IEEE Transactions on Pattern Analysis and Machine Intelligence*.

[16] Gonzalez R (2007). *Digital Image Processing*.

